# Hiding in Plain Sight: A Case of Perinephric Abscess Diagnosed by POCUS

**DOI:** 10.24908/pocus.v5i2.14430

**Published:** 2020-11-18

**Authors:** David Haughey, Tai Truong

**Affiliations:** 1 Dartmouth-Hitchcock Medical Center Lebanon, NH; 2 Dartmouth-Geisel School of Medicine Hanover, NH

**Keywords:** Renal, Kidney, Perinephric abscess, urinoma

## Abstract

An 87 year old male with obstructive uropathy was initially diagnosed with acute kidney injury (AKI), a new renal mass and hydronephrosis. When transferred to a facility with a hospital medicine POCUS program, the renal mass was correctly identified as a perinephric abscess, which was percutaneously drained leading to resolution of AKI and the underlying infection. Renal POCUS is readily taught via brief educational interventions and empowers providers to identify common (hydronephrosis) and uncommon (perinephric abscess) renal pathology at the bedside.

## Case File

An 87 year old male with past medical history significant for metastatic prostate cancer complicated by obstructive uropathy necessitating right percutaneous nephrostomy tube placement and stage-III chronic kidney disease (baseline serum creatinine ~1.5 mg/dL) presented in transfer from another hospital with gross hematuria and dysuria. His right nephrostomy tube had been placed seven months prior to admission. At some point during the interval period, the right nephrostomy tube was removed. Two days prior to admission, he experienced subjective fevers and generalized weakness. One day prior to transfer to this hospital he had hematuria and dysuria and was sent by ambulance to another local hospital. He was found to have a serum creatinine of 5.6 mg/dL and a urinalysis concerning for urinary tract infection. A bedside renal ultrasound done at that facility by a non-radiologist provider reportedly showed right sided hydronephrosis and a right upper pole renal mass. The report and images from this study were not available for review at the time of transfer. He received levofloxacin and a Foley catheter was placed. He was then transferred to this hospital. 

**Figure 1:**
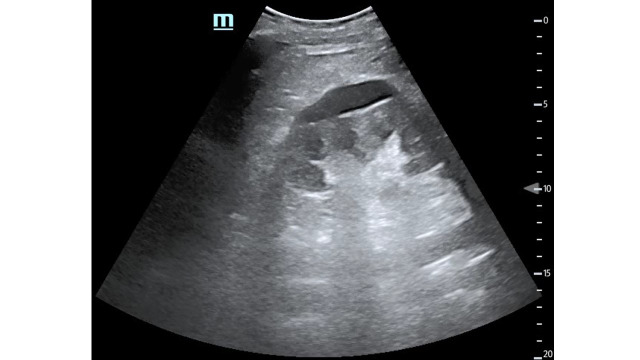
Right kidney in long-axis with perinephric abscess.

**Figure 2:**
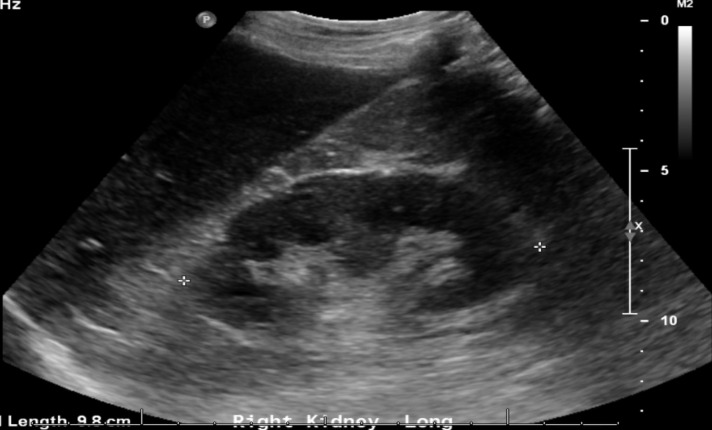
Right kidney in long-axis, after drainage of perinephric abscess.

On admission, vital signs were within normal limits. His abdomen was non-tender to palpation without palpable masses. The site of the prior right nephrostomy tube site was unremarkable. A Foley catheter was draining clear yellow urine. Laboratory work-up was significant for leukocytosis (28.2 x 10^3^/µL), blood urea nitrogen 80 mg/dL, and serum creatinine 4.85 mg/dL. Urinalysis was significant for >100 white blood cells and red blood cells and a “few” yeast per high powered field. On arrival, renal point of care ultrasound was done. The left kidney had no structural abnormalities. On the right, a perinephric anechoic fluid collection was seen, no hydronephrosis was present (Figure 1, online Video S1). Interventional radiology (IR) ultrasonographically confirmed these findings. Under fluoroscopy, IR injected contrast via the prior nephrostomy tube tract and contrast reached the perinephric fluid collection. A percutaneous drain was inserted into the fluid collection with return of ~30 mL of purulent fluid. Blood cultures grew Pseudomonas aeruginosa and Candida glabrata. Creatinine from the fluid collection was 4.2 mg/dL (serum creatinine was 4.3 mg/dL). The perinephric fluid collection grew Escherichia coli. He was started on micafungin and cefepime. One day later, repeat ultrasound showed resolution of the anechoic fluid collection (Figure 2). Creatinine returned to baseline and the patient was discharged on fluconazole and cefepime to complete his treatment course. He was seen two weeks after discharge in clinic and was doing well, at which time the drain was removed. 

Among patients requiring nephrostomy tube placement for malignant obstruction, up to 3.8% develop post-procedural acute pyelonephritis 1. Although uncommon, trauma secondary to nephrostomy tube placement can lead to urinoma or abscess formation. A urinoma can become secondarily infected and result in abscess formation; an abscess can also form secondary to bacterial translocation from the external environment to the kidney, via the nephrostomy tube 2. Urinoma and perinephric abscess appear as anechoic fluid collections on ultrasound. Prompt diagnosis expedites percutaneous drainage which can prevent abscess formation and/or progression of infection 3. Delayed recognition of abscess formation is associated with increased risk of mortality 4. The differential diagnosis for perinephric pathology with an anechoic appearance on ultrasound also includes perinephric hematoma, lymphangioma and pancreatic pseudocyst, whereas a heterogeneous echotexture can be seen in renal cell carcinoma, angiomyolipoma, lymphoma and liposarcoma. Chronic perinephric abscesses may have a heterogenous echotexture and thus be mistaken as a tumor and inappropriate over-gaining may compound this problem 5, both of which are potential explanations for initial misdiagnosis in this case. 

Many hospitals, particularly rural^6^ and community-based 7, do not have routine availability of radiology performed ultrasound. Patients at these facilities face delays until ultrasound is available or must be transferred to a hospital with ultrasound capability. Delays in diagnosis of urinoma or perinephric abscess prolong time to definitive treatment and increase the risk of adverse outcomes. Multiple institutions have successfully implemented brief training courses for novice POCUS practitioners to learn renal ultrasound 8. 

## Conflicts of Interest

None declared.

## Supplementary Material

Video S1 B-mode, right kidney in long-axis with perinephric abscess.
